# Applying systems thinking to knowledge mobilisation in public health

**DOI:** 10.1186/s12961-020-00600-1

**Published:** 2020-11-17

**Authors:** Abby Haynes, Lucie Rychetnik, Diane Finegood, Michelle Irving, Louise Freebairn, Penelope Hawe

**Affiliations:** 1 The Australian Prevention Partnership Centre, Sydney, Australia; 2 University of Sydney, Menzies Centre for Health Policy, Sydney, Australia; 3 University of Sydney, School of Public Health, Institute for Musculoskeletal Health, PO Box M179, Missenden Road, Camperdown, NSW 2050 Australia; 4 University of Sydney, School of Public Health, Sydney, Australia; 5 University of Notre Dame Australia, School of Medicine, Sydney, Australia; 6 Morris J. Wosk Centre for Dialogue and Department of Biomedical Physiology & Kinesiology, Simon Fraser University, Vancouver, Canada; 7 ACT Health Directorate, ACT Government, Canberra, Australia; 8 O’Brien Institute of Public Health, University of Calgary, Calgary, Canada

**Keywords:** Systems thinking, Knowledge mobilisation, Public health, Policy-making

## Abstract

**Context:**

Knowledge mobilisation (KM) is a vital strategy in efforts to improve public health policy and practice. Linear models describing knowledge transfer and translation have moved towards multi-directional and complexity-attuned approaches where knowledge is produced and becomes meaningful through social processes. There are calls for systems approaches to KM but little guidance on how this can be operationalised. This paper describes the contribution that systems thinking can make to KM and provides guidance about how to put it into action.

**Methods:**

We apply a model of systems thinking (which focuses on leveraging change in complex systems) to eight KM practices empirically identified by others. We describe how these models interact and draw out some key learnings for applying systems thinking practically to KM in public health policy and practice. Examples of empirical studies, tools and targeted strategies are provided.

**Findings:**

Systems thinking can enhance and fundamentally transform KM. It upholds a pluralistic view of knowledge as informed by multiple parts of the system and reconstituted through use. Mobilisation is conceived as a situated, non-prescriptive and potentially destabilising practice, no longer conceptualised as a discrete piece of work within wider efforts to strengthen public health but as integral to and in continual dialogue with those efforts. A systems approach to KM relies on contextual understanding, collaborative practices, addressing power imbalances and adaptive learning that responds to changing interactions between mobilisation activities and context.

**Conclusion:**

Systems thinking offers valuable perspectives, tools and strategies to better understand complex problems in their settings and for strengthening KM practice. We make four suggestions for further developing empirical evidence and debate about how systems thinking can enhance our capacity to mobilise knowledge for solving complex problems – (1) be specific about what is meant by ‘systems thinking’, (2) describe counterfactual KM scenarios so the added value of systems thinking is clearer, (3) widen conceptualisations of impact when evaluating KM, and (4) use methods that can track how and where knowledge is mobilised in complex systems.

## Introduction

Knowledge mobilisation is concerned with generating robust and useful knowledge and facilitating its movement and use in arenas where it can do most good [1]. This knowledge is often informed by diverse sources and is shared multi-directionally [2]. While knowledge mobilisation is well recognised as a vital strategy in efforts to improve public health, the ways in which it is theorised and operationalised vary widely, reflecting influences from different disciplines and paradigms [3, 4]. We make the case that systems thinking should be one of these influences. Systems thinking adds value because it offers productive ways of understanding and working with the multiple complexities that knowledge mobilisation strategies face. These include: 1. *“Wicked”* intractable public health problems that have multiple interacting causes and are characterised by uncertainty and conflicting values and views [5–7]; 2. Messy policy and practice environments which are constrained by competing demands and expectations, politicised decision-making and uncertainty [8, 9]; and 3. The dynamic nature of the open systems within which policies and programs are implemented [10–14]. Health is a property of many such systems including education, transport, the environment, housing, food systems, welfare systems and the economy [15]. Efforts to mobilise knowledge in public health policy and practice must take account of this complexity [5, 16–19].

Systems thinking has gained traction as a valuable approach to tackling complex problems as it offers concepts, tools and frameworks that have the potential to strengthen and complement knowledge mobilisation practice and research [20–22]. However, there is a long way to go in exploiting its full potential [23]. This commentary seeks to investigate how systems thinking changes the ways in which knowledge mobilisation is conceptualised and operationalised. We argue that systems thinking can do much more than enhance knowledge mobilisation: it can transform perceptions of what knowledge is, how it is created and valued, and how it is (and could be) used. Our aim is to move beyond description and theory to identify practical strategies, including examples of how systems approaches have been applied. We lay the groundwork by first offering an overview of the relationship between knowledge mobilisation and systems thinking, and then exploring three questions:

1. How can systems thinking advance knowledge mobilisation?
2. What does systems-informed knowledge mobilisation look like in practice?
3. What’s next for advancing systems-informed knowledge mobilisation?

**Fig. 1:**
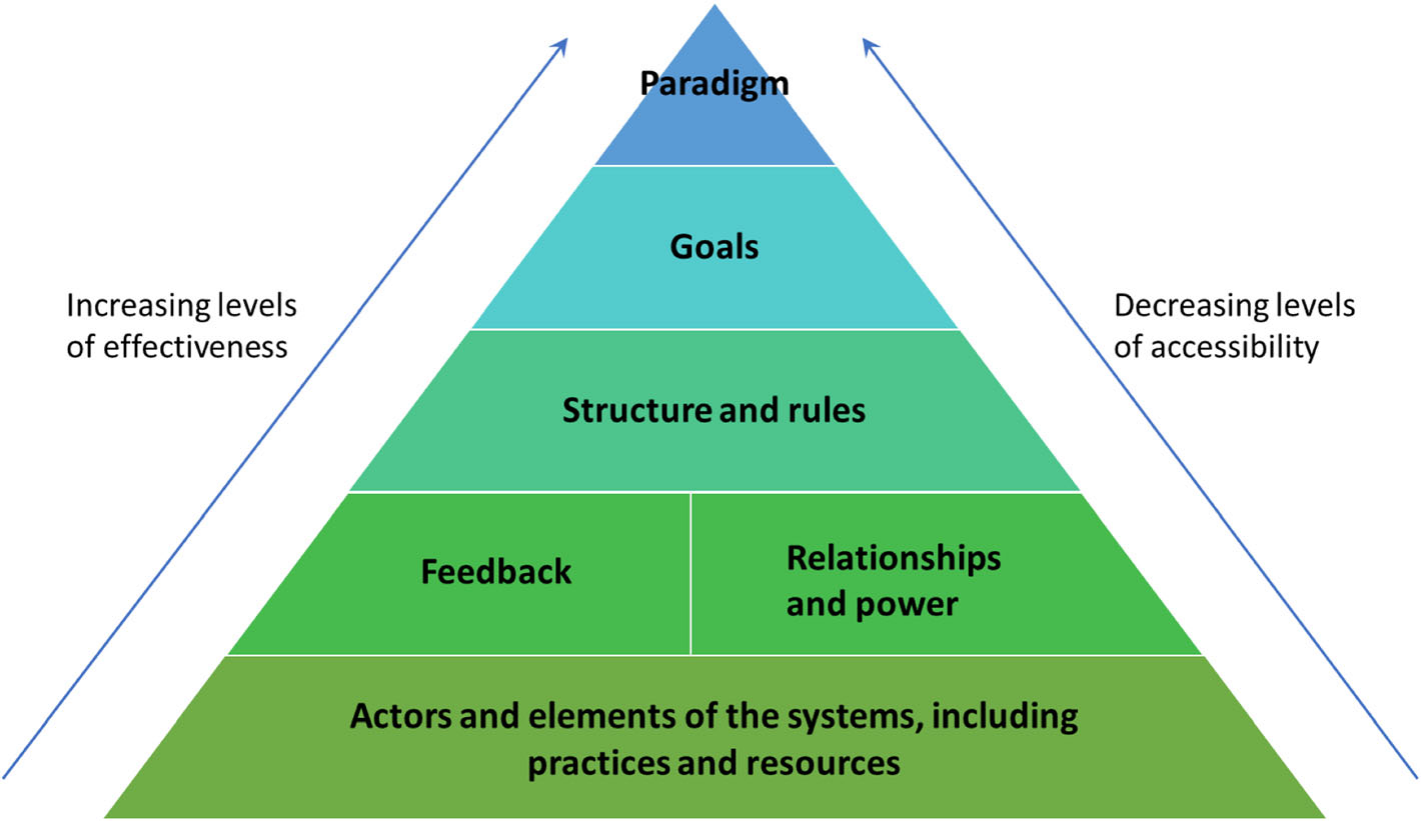
Leverage points for changing complex systems Adapted from Finegood, Malhi and colleagues [47, 48, 148] and Kania et al. [45]

We introduce key concepts, including frameworks for (a) understanding knowledge mobilisation (KM), based on eight empirically-derived archetypes of KM practice [3], and (b) using systems thinking to leverage change in complex systems (Fig. 1). We then describe how these frameworks interact (Table 1)—providing guidance for operationalising the ideas—and draw out some learnings.

**Table 1:** Overview of how systems-focused strategies and intervention points intersect with knowledge mobilisation practices^a^

Knowledge mobilisation archetypes	LEVEREGE POINTS FOR CHANGING COMPLEX SYSTEMS					
	How can we change … PARADIGMS (mental models)?	How can we change … GOALS (system function & aims)?	How can we change … STRUCTURE and RULES?	How can we change … RELATIONSHIPS and POWER?	How can we change … FEEDBACK?	How can we change … ELEMENTS (e.g. actors, practices, resources)?
**1. Producing and disseminating knowledge**	Knowledge producers must first reconcile their own paradigms with a ‘systems lens’, using methods for analysing and responding to complexity [14, 24, 25] Complex problems are multifaceted: embrace methodological and disciplinary pluralism [24, 26] Critique what knowledge is valued and most fit-for-purpose. An evidence-base for systems change is crafted differently to an evidence-base for biomedical interventions [2, 27, 28] Question universal hierarchies of evidence [28]: do not automatically privilege a particular interpretation of rigour if it is at the expense of *“ecological fit”* (local relevance and applicability) [29, 30] Use co-production to build in relevance, applicability and knowledge translation. It helps participants’ reflect on worldviews and frames of reference [28, 31]	Articulate the goals of collaborative knowledge production as part of wider mobilisation efforts. Including whether and how they fit with the goals of the system – leaders are in the strongest position to make this impactful [32] Consider *“multisolving”* approaches (also known as double-duty or triple-duty actions) that produce knowledge targeting two or more systems problems simultaneously [33–35] From the outset, strive to make research *useful* to policymakers and/or practitioners. Many argue that research is not ‘lost in translation’ but ‘lost before translation’ [36]. Researchers need to understand policy/practice challenges before trying to produce knowledge that influences them [37]	Develop structural and governance supports for cross-sector collaboration, co-production and engaged scholarship which includes those who are affected by the problem and those who are in a position to do something about it: this will produce more relevant and applicable knowledge [14, 38–40] [41] Embed accountability structures such as data-sharing agreements, explicit and flexible roles and deliverables, group decision-making, and distributed governance [42, 43]. Fund knowledge production that tackles problems systemically and uses systems-sensitive methods [44–46] Be conscious of what levels of the system you are targeting and shape knowledge production accordingly [45, 47, 48]	Ask whose knowledge is being privileged and with what impacts. Addressing power may require broadening the boundaries of participation and using methods such as co-production and participatory and action research [49, 50] Develop inclusive mechanisms for deliberation with extra support for those who are structurally disadvantaged [50–53]. Mechanisms can function at all level so consider how to support individuals, but also how structures and processes can be changed to optimise inclusion. Models for evaluating public involvement in research may help assess how stakeholders are involved in and influence co-produced knowledge [28]	Provide clear and accessible preliminary findings to participants and incorporate their views in final papers and reports [39] Explain any views that are at variance with researchers’ findings Collaboration to resolve complex problems involves iterative action, reflection and deliberation, sharing ideas and experiences as well as formal knowledge: build this into your process goals and timeline [28, 51] Engage stakeholders in interpreting and strategically communicating knowledge, e.g. blend community stories with epidemiological data to educate the media and other stakeholders about human costs and contextual realities [20, 33, 54, 55].	Direct resources to facilitate systems learning, capacity development and co-production [56, 57] Use creative strategies to communicate with and build systems thinking capacity among community partners and other stakeholders [58] Learn about and *from* the knowledge context [14, 59] : techniques include concept (or systems) mapping [60, 61], user journey mapping [54], participative dynamic systems modelling [62, 63], and social network analysis [24, 25, 64]
**2. Brokering one’s own research** **and** **3. Brokering bodies of research** Ideally, brokering will focus on syntheses (integrated bodies of knowledge) rather than individual studies thus, in practice, Archetype 2 should be embedded in Archetype 3.	Treat brokering as a long-term, relational process, not a ‘stage’ that follows knowledge production [65] Broaden the concept of ‘evidence’ in brokered syntheses to include diverse information and ideas, including from local contexts, that can shed light on problems and possible solutions [4, 66] Start with what matters to end users: knowledge is imbued with social meaning and its value depends on these meanings [27, 67] Brokering should not focus on transferring knowledge products, it is a form of change agency [68]	Shift the KM goals from bridging gaps to blurring the boundaries between groups and bringing them together [69] Knowledge brokers need to understand and find a ‘fit’ with organisational culture and goals. They must become credible insiders [70] [68] Aim to facilitate experiential learning and goal-orientated engagement with research rather than disseminating research products [71]	Embed knowledge facilitators within organisations to support engagement with research, collaboration, capacity development and organisational change [72] Create cross-sector training opportunities and secondments for researchers, practitioners and policymakers [72] Brokers should be embedded in organisations with strong support from leaders and adequate flexibility and resources [68]	Develop & resource credible trustworthy local champions who can influence how people engage with different forms of knowledge [17]. Knowledge brokers can support organisational knowledge orthodoxy or disrupt it: lessen constraints by placing brokers in senior roles, forming multidisciplinary brokerage teams, and linking brokers across different levels and parts of a system [73, 74] Ideas often have more impact than formal knowledge products [75]. This requires dialogue and relationships. Establish mechanisms to build trust and rapport between stakeholders [65]	Build a learning organisation that is engaged in continuous dialogue about how different forms of knowledge should be valued and used [17, 76] Create channels for communication and forums for networking and collaboration [77] Demonstrate that you are listening and learning, and changing your practice accordingly. Model reflective practice [2, 31, 45] Build shared vocabularies using local terms [62, 78]	Build capacity of individuals and organisations (via coaching, mentoring and interactive and applied learning) to produce, critique and use knowledge [2, 43, 57] Co-develop models and maps that can act as ‘boundary objects’ in stakeholder dialogues [58, 62, 79] Forge alliances with leaders who can influence the ways that knowledge is used in their organisations and networks [68]
**4. Advocating research**	Identify current mental models and ‘frame’ communications strategically to target them. Where appropriate, work to challenge mental models using narrative approaches that reframe concepts (i.e. shift blame or reconceptualise legitimacy) for greater impact [45, 80] Consider which beliefs are ‘core’ and which may be more amenable to change [81] Leverage ‘shocks’ or windows of opportunity in which societal foci, attitudes and values can be more malleable [80, 82]	Seek to understand and influence the outcomes that matter to key players in the system [31, 81, 83] Articulate a compelling vision of change for advocacy allies [80, 81, 84] Consider *“multisolving”* approaches (see Archetype 1)	Learn about the system: the stakeholders, key roles and organisations; who influences who; and who has power to make what decisions; who gains from different forms of knowledge? Consider what vested interest groups should be worked with, and which should not [45, 85, 86] Cultivate policy and practice ‘insiders’ who exert gentle persuasion with colleagues and ‘outsiders’ who create external pressure [3, 87] Identify ‘windows of opportunity’ and engage with real world policymaking rather than planning for a ‘rational’ orderly process [83]	Forge strategic alliances between communities, policymakers, not-for-profits & researchers to generate political pressure [53, 81]. Develop *“policy entrepreneurs”* who can navigate policy terrains, attract resources & influence powerful decision-makers [82] Consider ‘Who can act on this knowledge?’ & ‘Who can influence those who can act?’ [88] Call out & counter the influence of vested interests (& problem framings) that perpetuate social injustice & inertia [14, 85, 89] Strengthen civil society, support social movements and help marginalised groups have a voice [87]	Engage in *“policy learning”:* monitor shifts in the policy landscape and adapt accordingly, especially during implementation when policy contributes to positive or unintended outcomes [81] Develop media expertise and forge working relationships with on-side journalists who can reach mass audiences and impact public opinion [90] [87, 91] Use stories, metaphors, symbols and images to communicate findings powerfully and engage others in discussion [63, 82, 87]	Educate health researchers and practitioners in systems-informed advocacy techniques and position advocacy as a legitimate (vital) aspect of public health KM [80] *“Mobilise troops”*: engage members of the public in political activism and fund raising [81] Draw on different forms of knowledge strategically for different audiences and at different times [87]
**5. Getting research into practice (implementation)** **and** **6. Researching in practice** **(local learning and knowledge production as part of implementation)** From a systems perspective, these practices are iterative and entwined: implementation should contribute to and be guided by local learning and knowledge production.	‘Diagnose’ the context you are dealing with so you can select appropriate intervention and implementation strategies [92] Recognise that knowledge is not objective or universal: it evolves, and must be understood, within the context of practice [27] Systems interventions are not discrete projects: focus on emergent change rather than tangible short-term outputs [93] Move away from prescriptive practice models towards empowerment models [94] Complex problems may be shifted but are seldom fully ‘solved’ [95]: Forego the search for ultimate solutions and invest in a ‘positive error culture’. Outcomes and theories of change should be responsive. Trial and error learning and adaptation is key [44] Allow sufficient time to establish community-based interventions so that the knowledge being generated is easier to appreciate. Systems change takes time to embed and show meaningful results [96]	Consider how the system’s dynamics and history of change shape the current change trajectory [14, 16, 97] Find ways to tap into what drives people in *this* system. Focus on measures and outcomes that reflect local stakeholders’ interests [44] Develop shared goals and support practitioners to modify their practice so that they can own them: *“if people cannot take care of a problem, they won’t see a problem”* [94]. Use tight-loose-tight KM approaches where the purpose of the project and its outcomes are tightly specified, but its design and implementation are participative and loosely specified, so that they can be adapted locally for maximum effect [98–100]	Implement strategies flexibility, guided by principles and adherence to ‘form’ rather than fidelity to static rules: *“help it happen”* not *“make it happen”* [101–103] In scaling up, focus on adaptation, interdependencies and stakeholder sensemaking, and responsivity to unintended consequences [11, 104] Leaders should use strategies and facilitate conversations that build organisational learning and foster innovation and positive deviance [92] Create flexible rules (policies, guidelines) that allow the system to evolve. These usually incorporate learning into the management process [32] Create opportunities within organisations for people to take on new roles and forge new relationships aimed at mobilising knowledge [64]	Share leadership among a distributed group of champions and influencers at different levels who model desired values and practices [43, 105, 106] Build ownership through collaboration with service providers. Work with local champions to diffuse ideas, get feedback and adapt knowledge and implementation strategies for local ‘best fit’ [17, 66, 94, 104] Build on innovations that emerge from service delivery and have been shown to work well for local communities and organisations [29] Support service users (especially those who are seldom heard) to participate in local knowledge generation (see the ‘Addressing power imbalances’ section of this paper for examples) Build indicators and accountabilities in collaboration with frontline staff [107]	Help knowledge inform action: use existing feedback loops to promote dialogue and provide updates, and create new feedback channels where needed [93] Invite discussion and convey feedback in locally meaningful ways [15, 20]. Use stories to surface tacit practice knowledge and explore ambiguities [108] Use reflexive methods that attend to system dynamics and direct process learning back into change efforts, e.g. ‘learn and adapt’ cycles, continuous improvement, developmental evaluation and researcher-in-residence models [109, 110]. In these approaches learning itself is a feedback loop [44] Resist *“drift to low performance”*: promote positive performance feedback and articulate underpinning values [32] Watch for windows of opportunity in policy implementation and capitalise on shifts in politics and political will [83, 111]	Use research and evaluation methods that capture the views and lived experiences of key stakeholders, e.g. co-production, action research [54, 58, 112] In many areas of complex policy and practice there is no universal ‘best practice’, only emergent practice [113]. Work towards ‘best fit’ which focuses on optimal adaptation and reach in your context [114] Direct funding and other resources at learning-orientated implementation [44]. Focus on workforce capabilities (responsive capacities) rather than competence (fixed skills) [115] Use tools that can visualise non-linear dynamics, including complexity-sensitive logic models [58, 116, 117]. Don’t discard robust behavioural frameworks [118] but note that interventions focused exclusively on individual elements may be less effective in changing systems [119]
**7. Fostering networks** Systems-informed KM interventions can impact formal and informal networks. Formal networks are collaborations of people or organisations working for a common purpose. Informal networks are everyday ‘who-knows-who’ relationships.	Deliberately form connections across paradigms, disciplines and epistemic communities [42, 86] Do not aim for unified consensus but for ways of understanding and bringing together diverse perspectives [27] Utilise network science and other systems-sensitive methods to map and analyse developments [39, 120] Both formal and informal networks can influence decision-makers. Employ the communication strategies described in archetype 4, above [85]	Develop and articulate a shared understanding of the underlying goals of any formal network and a clear vision of what they are working towards [43] Critique the boundaries within which formal network members are identified: are they wide (inclusive) enough to address the network’s goals? [49, 50]	Structural connectivity will affect functional connectivity: agree on structures and systems for supporting formal networks (see no. 1 above) [42, 43], including clear roles and rules for decision-making [16] Where possible, articulate and address the structural (institutional, epistemological and political) boundaries that impact knowledge sharing and use in formal networks [22] Consider how networks often sit within and are linked to other networks: can these wider connections be used to maximise KM? [16]	Identify and work with influential connectors to foster and strengthen relationships [42] Develop mechanisms for inclusivity that give all members a voice [43] Invest in the quality of relationships as much as project delivery. Mutual trust and respect are fundamental [44]	Build a community of practice that learns together and can influence change in multiple arenas [84, 121] Effective formal networks continuously improve through reflecting and acting on feedback [16]. Continue to review and amend linkage and governance mechanisms, communication, working processes, and KM strategies and goals [42] Ensure formal network members are well-informed about early wins in the network’s goals and have a say in how next steps should proceed [43]	Develop capacity building initiatives that can help members to participate fully within a collaboration [42, 122, 123] Provide platforms for meeting and sharing knowledge (technological and face-to-face), and informal networking [40, 123] Evaluation strategies suited to capturing the effects of social networks include: interviews, spidergrams, social network analysis (especially useful for studying and visualizing complex structural ties over time [23, 64]), dynamic systems modelling and Photovoice/Videovoice techniques [39].

^a^This table omits the eighth archetype— Advancing knowledge mobilisation—as that is the focus of the whole table and this paper

## The relationship between KM and systems thinking

### What is knowledge mobilisation for public health?

Knowledge mobilisation refers to a broad set of activities that,

*“… help move research results into society, as well as bring new ideas into the world of research. From knowledge-brokering and outreach, to more effective dissemination through new technologies, to the ‘co-creation’ of knowledge, these processes help ensure that public investments in ... research have the greatest possible impact—intellectually, socially and economically.”* ([1]:12)

In public health, knowledge mobilisation (KM) focuses on impacting public policy as well as health service management and practice. There is widespread agreement that research-infused knowledge should inform policy and practice but systems and structures are often very poor at facilitating this. As Boaz et al argue, *“KM is in everyone’s interests and no-one’s job description, and everyone blames everyone else for its absence”* [124].

There is also debate about what forms of knowledge should be mobilised, how and with what aims. Echoing the changing discourse about evidence-based policy, the dominant conceptualisation of how knowledge is shared has moved from a linear model towards multi-directional and complexity-attuned approaches where knowledge is produced and becomes meaningful through social processes [3, 27]. The literature on Mode 2 knowledge production [125] and work by Graham, Ward, Nutley, Holmes and others (e.g. [2, 4, 20, 38, 126, 127]) emphasises that knowledge is not independent of systems, processes or people and that context, co-design, power relations and different ways of knowing are key constructs. The evolution of terminology (e.g. transfer, translation, exchange, diffusion, integration and mobilisation) illustrates this well [2] (although the proliferation and inconsistency of terminology has also been noted [128]). Similarly, what is being mobilised is increasingly conceptualised more broadly as *knowledge* rather than *research* or *evidence.* This recognises that policy and practice are necessarily informed by a fusion of locally interpreted information, ideas and values from multiple sources, only some of which stem from academia [129, 130]. We use the term *knowledge mobilisation* here because it seems the most fluid and inclusive option for capturing what occurs in real-world settings. The influence of, or overlap with, systems thinking can be seen in this evolution but, as we will show, there are further contributions that systems approaches can make to inform or reconceptualise contemporary understandings of KM practice.

### What is systems thinking?

Systems thinking in public health is a broad conceptual lens informed by a multidisciplinary body of theories, tools and methods [32, 49, 97, 131]. It posits that the world is comprised of complex systems which are dynamic, interconnected and evolving, and cannot be controlled, so they must be better understood if we are to effect desired change [10, 16, 132, 133].

A system is a perceived collection of interrelated but independent parts that are linked by a common purpose and, through their interactions, function as a whole (see Box 1.). It includes the range of actors, activities and settings that appear to have direct or indirect influence on (or be affected by) a given situation [112, 134]. Systems that impact public health may include communities; coalitions and networks; school, hospitals and other government services; policy agencies that work at local, state and national levels; and broader economic and political systems that impact on how wealth and health are created and distributed.

Systems thinking makes a crucial distinction between *simple, complicated* and *complex* phenomena. Where systems (and the problems they produce) are simple or complicated they are relatively linear, deterministic and stable with few interdependencies. In these relatively ‘knowable’ situations, planned KM strategies with fixed outcomes may be most appropriate. Where they are complex, however, they are non-linear and dynamic, adapting unpredictably in response to feedback and thus often undermining ‘logical’ efforts to bring about change [64, 119]. In these situations, systems approaches to KM are likely to be most valuable because they invite a multi-perspective, emergent view of the phenomena.

Systems thinking has an increasingly strong presence in the public health literature with near-exponential growth in recent years [145]. For many, it offers valuable perspectives, tools and strategies for better understanding problems in their settings and, potentially, for strengthening policies so that they are more inclusive, effective and resilient [14–17, 24, 29, 32, 146–148]. However some authors note that the contribution of systems thinking to improving public health quality or intervention effectiveness is not yet clear or convincing, suggesting that it may be a passing fad offering little more than a compelling metaphor [138, 149–151]. This may be, in part, because it is currently underutilised and under-studied [133].

### Using systems thinking to leverage change in complex systems: a framework

Systems thinking encompasses socio-ecological models within which health problems and solutions are conceptualised as multi-level phenomena involving individuals, groups, organisations and wider systems at sector and societal levels [4]. But it also draws attention to the structures, interactions and forces that operate within and across social layers and shape causality [148]. Systems approaches focus on patterns of inter-relationships, rather than independent forces, as the leverage points for intervention [76, 132, 152].

Malhi and colleagues [47] (who adapted work by Meadows [32, 132]) and Kania et al.,[45] suggest six key areas or leverage points to target when applying a systems approach to organisational change. Each of these areas raises questions for practicing KM:

#### Paradigm

What are dominant mental models in this system? What assumptions are held about how the system works and should work? How can these values and beliefs best be leveraged (or challenged) in efforts to mobilise knowledge?

#### Goals

What do people believe are the key aims of this system, its overall purpose? Are the espoused goals congruent with what really drives people? In what ways do current goals and past achievements align with the goals of KM? How can ‘goals of goodness’ (aiming for positive practice) be enhanced in this context?

#### Structure and rules

What are this system’s organising foundations (including infrastructure, and rules such as policies and guidelines)? How do they shape knowledge practices and opportunities? How do past experiences impact the present and possible futures? What changes in structure or rules might best support the system to self-organise productively in relation to KM?

#### Relationship and power

What types (and quality) of connections are there within and between people and institutions? How do roles and decision-making powers influence change trajectories? What opportunities are there to create new roles and relationships, leverage existing relationships, and address power imbalances?

#### Feedback

What forms and channels of information help patterns of change to remain stable in this system? Which cause patterns of change? How can existing feedback channels be used (or new ones created) to generate engagement with KM efforts, and to sustain desired change?

#### Actors and elements

What people, practices, resources, and physical elements comprise this system? Interventions that target discrete parts of a system are often ineffective in creating system-wide change (or require many actions at this level) so is this the most effective strategy? If so, what else needs to be done?

As Fig. 1 indicates, these areas are conceptualised as a hierarchical framework in which change is harder to achieve at the upper levels but, if successful, is likely to be more transformational. Systems change will usually require action at multiple levels, often simultaneously, but this framework can guide decisions about where best to focus efforts and what sort of strategies might be most successful.

In the next sections we explore what systems-informed KM might look like and offer suggestions as to how that could be operationalised in public health policy and practice.

### What is the current relationship between systems thinking and knowledge mobilisation in public health?

Systems thinking has a growing presence in KM discourses [3, 20]. Commentaries recognise that not only are public health problems and solutions shaped by the forces of complex systems, but so is the practice of KM itself. From this perspective, KM is also emergent, highly relational and profoundly context dependent [62]. A systems approach to KM may include identifying who (or what) has the power to influence which forms of knowledge are valued and used, and recognising that KM efforts can create ‘push back’ from a system, resulting in unexpected and counterintuitive outcomes [87]. As we argue below, systems thinking can significantly recalibrate the scope of strategies and targets associated with mobilising knowledge to inform public health policy and practice.

Systems thinking yields strategies that improve the value, reach and impact of knowledge [20, 62, 134, 153]. This includes knowledge about the co-benefits of policies and programs that address the complexity of entangled and syndemic public health problems [10, 32, 33]. Empirical studies indicate that intervention outcomes are positively associated with strategies that address the complexity of processes and conditions they are trying to improve. For example, studies of 124 interventions tackling congestive heart failure, type II diabetes and hospital readmission rates found greater effectiveness when the interventions targeted two or more characteristics of complex systems (including interdependencies between people, resources, processes and structures), and when they allowed the implementation to evolve responsively over time [141, 154, 155]. These intervention designs were congruent with systems principles but not explicitly informed by them. This suggests that systems ideas may be having an ‘enlightenment’ effect [156]: seeping into policies and programs by influencing underlying mental models rather than directing conscious choices.

System thinking has inspired whole-school level co-production approaches to primary prevention. Cluster-randomised controlled trial methods have shown these to have greater effect sizes in reducing adolescent smoking and drug and alcohol use than conventional (health curriculum) methods [157, 158].

Other empirical studies indicate that systems thinking can significantly improve leadership performance and organisational efficiency [159], and is integral to effective project management [160] and organisational crisis responses [161]. Systems approaches can also optimise specific practices such as improving the use of organisational level knowledge [17], co-producing research, network formation, fostering ‘bottom-up’ innovation, implementation and scaling-up [142]. Single studies have found that local solutions implemented flexibly by frontline staff, and supported by reflexive learning and discussion, improved patient safety in five hospitals [94]. Willis et al. describe how systems approaches underpinned a large-scale healthcare transformation tackling improvements in surgical redesign, primary care and quality improvement [98]. Systems thinking has also been used in process evaluation to develop explanatory understandings of complex relationships between individual interactions and health organisation structure and functions [162].

Despite this growing body of evidence about the value of systems approaches for addressing complex problems, and calls for applying system thinking to KM in public health, the actual practice of KM has not necessarily changed [71]. For example, a recent global survey of 106 research organisations found that most of their KM strategies focused on ‘pushing’ their own research (a strategy the authors call *“disseminate and hope”)* with very little use of strategic ideas from the literature that *“take account of competing definitions of knowledge, the internal and external contexts, the parties involved, the organisational factors and the political dynamics”* [153]. This may be in part because dominant KM and implementation frameworks do not currently take account of core concepts in systems thinking such as unpredictability and emergence, dynamism and the need for local autonomy [142, 163]. Further, the literature on applying systems approaches to KM in public health policy and practice has few operational models or in-depth case studies, and minimal practical guidance on how it should be done [3, 134]. We seek to address this gap.

## 1. How can systems thinking advance knowledge mobilisation?

Davies and colleagues [3] describe eight archetypes of KM practice that were derived inductively from an extensive literature review and empirical investigation of practices used in organisations that aim to mobilise knowledge for policy and/or practice (including website review of 186 international agencies, in-depth interviews with agency leaders, a web survey and deliberative workshops with stakeholders). These archetypes integrate the patterns of KM assumptions, activities, configurations and rationales observed across the agencies and can be used to map the breadth of KM and its basic architecture [3]. Impacting policy is considered an outcome rather than a practice in its own right.

We now explore the contribution that systems thinking makes to each of these archetypal practices, and reflecting on how a systems approach might further extend, strengthen or transform them.

### Archetype 1: Producing knowledge

*This archetype includes the production and dissemination of research-based products such as empirical research papers, systematic reviews, research summaries and syntheses and web portals. Its focus is on instrumental, knowledge-driven problem solving*.

Traditionally, knowledge production has been viewed as an exclusively scientific endeavour, separate to the knowledge transfer processes that followed it. The dominant scientific mode (which privileges internal validity, objectivity, reliability and generalisability) has produced robust knowledge that has advanced many areas of public health, but it has some important limitations for addressing policy and practice problems where complexity is a defining characteristic [30, 130, 164–166].

With its focus on interaction and dynamism, systems thinking demands that knowledge production grapples with real world contexts [144]. It is methodologically pluralistic and creative, designing new methods to address complexity [23]. It also integrates theories, ideas and views from multiple sources so is usually transdisciplinary and collaborative [24, 62, 71]. For example, the emphasis on ‘What works?’ is shifting to take account of much broader questions exploring social impact, causality and equity [167], and how best to include the voices of people whom this knowledge is intended to benefit [124]. Systems thinking (in common with some other approaches, e.g. participative action research [168]) makes this explicit by asking how knowledge production and use reflects community needs and attends to local and institutionalised values and power inequities [45, 50, 89]. This expands and changes what we focus on, how we conduct the research, how it is integrated with values and other forms of knowledge, how we try to implement it, and who is involved. Ultimately, it reconceptualises what we mean by knowledge and by “good evidence ”[167].

### Archetypes 2 and 3: Brokering and intermediation

*These two archetypes describe relational models for promoting research flow and use. Key activities include tailored dissemination, education, and the creation of interactive spaces for researchers and policymakers/practitioners. Archetype 2 focuses on brokering new local research, while archetype 3 focuses on brokering wider bodies of existing research.*

Intermediaries have a vital role to play in brokering and championing new knowledge and the strategies used to mobilise it within local contexts [17]. This is well established in the wider KM literature (e.g. [169, 170]). Systems thinking adds to this by emphasising that public health problems are the products of complex social, economic, political and institutional forces [10]. This encourages mobilisation efforts to break down silos, integrate different forms of knowledge, and facilitate its use synergistically across different layers and sectors of government [15, 24]. So, from a systems perspective, intermediary roles are less about ‘bridging gaps’ or packaging and ‘transferring’ research findings, and more about ‘blurring the boundaries’ between groups and enabling a more continuous process of knowledge exchange as part of everyday practice [69, 171]. This can involve building capacity, creating hybrid roles, harnessing local champions and communication channels, bringing people together, and developing shared vocabularies, knowledge and objectives. Several studies illustrate the benefits of collaboratively generated visual models and maps as ‘boundary objects’ that provide a focal point for such activities [58, 62, 79].

### Archetype 4: Advocating evidence

*This archetype describes proselytising for evidence-informed action in which interaction is central. Activities include education, social influence, forging alliances, and the use of incentives and reinforcements. Advocacy recognises that knowledge use is socially situated and so aims to reduce structural, organisational and cultural barriers to desired health outcomes.*

Public health advocates tend to be systems thinkers whether or not they use that term. They analyse systems to identify power structures, vested interests and leverage points; mobilise important stakeholders and the media to put pressure on these points; and ‘frame’ their communications to target and change prevailing mental models [90]. For example, the advocacy coalition framework recognises that political change is complex and uncertain, thus the need for *“policy learning”* and cross-system efforts to influence feedback loops, values and beliefs [81]. The anti-tobacco movement is notable for its success in denormalising smoking using complementary strategies to target legislation, public perceptions and industry misinformation [172]. Such approaches deliberately engage with multiple levels of systems as illustrated in Fig. 1.

### Archetypes 5 and 6: Getting research into practice and researching in practice

*Archetypes 5 and 6 describe hands-on support for local implementation, developing networks and building local knowledge capacities. Archetype 5 focuses on improving practice through the application of research from outside the organisation where change is sought. This may emphasise ‘transfer’ of explicit knowledge, but its adoption can also include local adaption and contingencies. Archetype 6 focuses on producing knowledge within the organisation. Key activities may include local learning, capacity development and co-production.*

KM has tended to be conceptualised as a staged activity with models that focus on rational cognitive processes and linear ‘transmission’ routes (e.g. pipelines, ladders [173, 174]). Increasingly however, the KM literature recognises that the use of knowledge is contingent, dependent on perceptions of credibility, legitimacy and political acceptability which are shaped by epistemological beliefs, ideology and changeable local conditions [175, 176]. As Nutley et al. argue,

… there is no simple answer to the question of what counts as good evidence. It depends on what we want to know, for what purposes, and in what contexts we envisage that evidence being used [130].

Systems thinking builds on these developments by introducing the concepts in Box 1 which have profound implications for development, implementation and evaluation of policies and programs. Interdependence, self-organisation and emergence means that leveraging change in one part of the system will lead to desired outcomes only if the right concurrent shifts happen in the system’s wider relationships and structures [112], and thus mobilisation strategies cannot always be planned and implemented with predictable effects [14, 136]. For example, from a systems perceptive, interventions will scale-up more effectively if they are conceptualised as provisional plans for action that work with variation across local contexts, striving to harness the self-organising and sensemaking capacities within intervention settings [11, 144]. This involves a ‘bottom up’ approach of working closely with stakeholders, fostering local champions and providing strategic feedback for ongoing problem-solving and adaption [104, 144].

A systems lens on archetypes 5 and 6 also changes how we understand and study implementation fidelity. Hawe argues that interventions are not replicated in the same forms across complex systems, rather, what gets transferred are the intervention’s core principles [29] or what some call its *“powerful ideas”* [177]. This means rejecting the traditional doctrine of precisely implementing intervention components (its form or surface structure) and instead focusing on fostering and evaluating local adaption that is consistent with the intervention’s theory of change (its function or deep structure )[29, 64, 101, 178, 179]. The contribution of systems thinking to this burgeoning *“science of intervention adaptation”* [178] can be to distil the essence of an intervention, design more fit-for-purpose methods to embed it, enhance its local effects, and help evaluate it [29]. This highlights a need for qualitative methods that can capture and use emergent knowledge generated in the adaptation process (e.g., what is the new knowledge about, for what purpose, in what form, used by whom and how?).

When intervention design and implementation are poorly integrated, interventions may be ill-suited to local populations and settings, resulting in implementers having no sense of investment or ownership, and a poor understanding of how to deliver their core functions [94, 180]. Collaborative problem-identification and intervention design (which includes those implementing and receiving the intervention), together with fostering local adaptation, are systems-informed responses to this enduring challenge [14, 105, 180].

### Archetype 7: Fostering networks

*This archetype describes the creation, development or facilitation of linkage and collaboration to shape and share expertise and increase the role of research-based knowledge. Knowledge from within, and external to, the organisation/network is used, and attention is paid to tacit and local knowledge. Knowledge production and/or dissemination is combined with social influence and facilitated interaction.*

The wider KM literature draws attention to the significant epistemic and cultural differences between the worlds of research, policy and practice [181–183], and to the detrimental effects of silos within health research itself [184]. Thus many agree that cross-sector and transdisciplinary networks, including communities of practice, are key to effective KM [26, 42].

Systems thinking adds to this by exploring how networks of people or organisations exchange information to work together, and how network structures change across time and new structures emerge. It highlights the benefits of operating both vertically (connecting layers of government and health systems) and horizontally (connecting sectors) [15, 59]. Strategies for fostering networks build on and ‘nudge’ existing connections and dynamics, and strive to develop feedback mechanisms for engagement and exchange [39, 56]. Viewing formal collaborations as networks also invites boundary critique: asking who and what will be included in the enterprise, and what this means for equity and the values (and ‘facts’) that are represented [49, 50, 185]. This goes well beyond a focus on disciplinary boundaries to consider the institutional, epistemological and political boundaries that impact on knowledge sharing and use [22]. Boundary critique requires explicit justification of choices and fostering participation by stakeholders who may be marginalised by existing structures and processes [50].

### Archetype 8: Advancing knowledge mobilisation

*This last archetype describes attempts to develop a coherent theoretical foundation for KM and to evidence it with robust empirical evaluation. The aim is to refine the field, build shared understandings, generate commitment to further study and reflexively apply “knowledge about knowing”.*

Systems thinking advances KM as a field by shining a lens on the complex, real-world contexts in which knowledge is produced, set in motion and used. For example, it tells us that KM contexts are interdependent and dynamic so that effective KM is likely to be multifaceted, iterative and adaptive. Consequently, these archetypes have fuzzy boundaries and should be employed complementarily depending on circumstances and goals. Systems thinking also goes beyond these archetypal practices. It promotes a paradigm that questions what knowledge is, how it should be generated, the contexts and processes in which it is used, methods for mobilisation, what impacts we can hope to achieve through KM efforts and how they should be evaluated [14, 56, 59, 144, 148, 163]. KM is no longer conceptualised as a discrete piece of work within wider efforts to strengthen public health, but as integral to and in continual dialogue with them [31].

The influence of systems thinking becomes more prominent as these archetypes progress. In the first three (producing knowledge, brokering local research and brokering bodies of research) research knowledge tends to be treated as an exemplary product that has some independent value. This construes KM as a *complicated* practice in which reduced barriers or enhanced enablers will facilitate knowledge use [186]. In the latter four archetypes, knowledge is positioned as more dynamic and imbued with value (or not) through a social process of negotiated sensemaking and local adaption. Here, context is foregrounded and knowledge is both generated and used by those with very different forms of expertise [187]. Values, rhetoric and tacit knowledge gleaned from personal and professional experiences are part of the mix [11, 144, 188]. These archetypes, therefore, tend to construe KM as *complex*. This highlights the existing influence of systems ideas but also suggests the need for a coherent systems-informed approach if KM is to achieve its potential.

## 2. What does systems-informed knowledge mobilisation look like in practice?

Table 1 presents an overview of how systems thinking can inform strategies and intervention points for KM. It explores the archetypal KM practices described above in relation to the systems change framework depicted in Figure 1 and the questions raised by KM in each area of the framework. The purpose is to provide practical guidance about the range of considerations that may be useful when planning, developing and implementing KM strategies for complex problems.

The ideas and recommendations in this table are not new; many of them are currently used by individuals and organisations, albeit variably and often without a systems label. The content is also not definitive; others will have different ideas and may challenge some of our assertions. Many of the concepts and strategies in the table have roots in other traditions and are thus not the sole property of systems thinking, but are potentially repositioned or given new force when KM is viewed through a systems lens [187]. It may be considered somewhat paradoxical to use a table to describe complex interdependent phenomena, but tables are familiar communication devices that enable information to be distilled efficiently. In this table, dotted lines between rows and columns indicate permeable boundaries and potential fuzziness in the distinction between concepts or even in which cell they should sit.

The strategies to support systems-informed KM as outlined in Table 1 should also be underpinned by a healthy humility that recognises the complexity and unpredictability of the systems within which public health problems are created and solved, and how the perspectives of different actors, including ourselves, are invariably limited [97]. This suggests that collaboration/co-production, empowering stakeholders and continual learning about the interactions between context and mobilisation activities are cornerstones of this work. As noted previously, such ideas are not exclusive to systems thinking, but they are elevated by it. We expand on this below with some examples of practical tools and application:

### Contextual understanding

Systems thinking moves the context into the foreground [187]. It demands that we understand what local needs look like, the meanings attached to different forms of knowledge, how it moves around the systems, and how knowledge is constrained or gains legitimacy, including key agencies, actors, systems structures and dynamics. This helps the development of contextualised theories of change, indicates possible leverage points and strategies, indicates where best to draw the system’s boundaries and aligns expectations with local realities [14, 59, 64, 66, 78, 112, 116]. It also raises questions about how knowledge claims can be established, e.g. what forms of interrogation about accuracy and authority are appropriate? This nudges the focus of KM strategies towards the more interactive and participatory activities associated with the latter archetypes.

In many cases, contextual understanding will require explicit systems analysis. Examples of systems analysis methods and tools include concept (or systems) mapping [60, 61], dynamic systems modelling [62], and social network analysis [24, 25, 64]. These approaches are often used as the starting point for stakeholders to come together for collaborative KM. When visual representations of systems are co-produced (e.g. behaviour-over-time graphs, causal loops, stock–flow diagrams and simulations) they support joint problem-framing and diagnosis, help identify solutions and motivate those involved to take action on proposed solutions [189].

### Collaboration and co-production

A central feature of the systems approach across all the archetypes is cross-sector deliberative collaboration (or co-production) with key stakeholders. This is demanding terrain. Challenges include the need for additional time and resources, and management of power inequities and conflicting values and priorities [190]. Collaboration is not always appropriate or feasible so other forms of participation may be preferable in some circumstances [41]. Some argue that collaborative research has the potential to harm reputations, relationships and research integrity [190]. Empirical studies have found that genuine co-production is hard to achieve in practice [191] and that partnerships can be plagued by distrust, inertia and antagonism [192]. There is little guidance about how best to constitute and support collaborations as (and within) dynamic systems.

Yet others report that collaboration (and closer relationships in general) can combat distrust between epistemic groups [193]. Collaborative relations provide critical nuance by eliciting stakeholders’ tacit, experiential knowledg e[191], increase the perceived relevance, applicability and legitimacy of knowledge thereby facilitating its use in policy and practice, and can increase accountability and investment by those who are best positioned to champion or facilitate strategies at the local level [16, 25, 28, 94, 143, 193–195]. The mechanisms for collaboration are relatively well understood [122, 123, 196] and there are many examples of where it has been used effectively for knowledge mobilisation. These include cross-sector partnerships to empower first nations people [51–53]; intra-organisational collaboration that generates ‘frontline ownership’ by healthcare providers [94]; co-developed equity indicators used by public health agencies to address social determinants of health [28]; cross-sector social learning platforms that have tackled health promotion, food security, campus health systems and tobacco control [39]; stakeholder co-production of a framework for natural experimental evaluation of a levy on soft drinks [197]; and a national partnership using systems science to prevent chronic disease [40, 123]. Bammer [41] describes tools for identifying levels of collaboration, for stakeholder analysis and knowledge synthesis, and for understanding value conflict and compatibility.

### Addressing power imbalances in how knowledge is constituted and legitimised

Systems thinking across all the KM archetypes also invites consideration of the insidious detriments of formal and informal power inequities, including the influence of vested interests [45, 50, 85]. We need to ask questions about how knowledge is constructed, whose knowledge is being mobilised for what purpose and whose gain, and what our own role is within that dynamic [2, 86]. Collaboration with those who are disempowered is essential; especially with people who have been structurally disadvantaged [51–53]. Examples at the micro level that illustrate how different perspectives (knowledge) can be brought to the table include paying for first class disability train tickets so that stakeholders with disabilities can participate in workshops [195], using empowerment techniques to support young homeless people to generate service design proposals [50], and linking community members with researchers in a buddy system [54]. At the meso levels, strategies may involve modifying roles or procedures within an organisation [198], establishing advisory bodies comprising members of disenfranchised communities [45], or using citizen juries, deliberative polls or boundary organisations and objects [167]. Beyond this, participatory action research methods are designed to retain the voice and control of those typically marginalised [199].

### Adaptive learning

To mobilise knowledge within self-organising, dynamic and unpredictable systems, we need to engage in continuous learning and adaptation to identify and nurture emergent local responses. This is exemplified in archetype 6 (researching in practice) but also applies to brokering knowledge, advocacy, implementation and fostering networks. The value of reflexive learning-in-practice is emphasised by Brown and Duguid [200] who differentiate between *modus operandi* (the way a task looks to someone working on it as it unfolds over time when *“many of the options and dilemmas remain unresolved”*) as opposed to *opus operatum* (the way a finished task appears in hindsight). *Opus operatum*, they argue, focuses on the task and glosses over the process of doing which is structured by changing contextual conditions. This means that as a work process becomes more complex, our post-hoc depiction of it increasingly obscures what actually needs to be done. Close attention to *modus operandi* is needed to understand how knowledge is (and can best be) generated, shared and used.

Adaptive learning requires observation and understanding of the flow-on effects and feedback loops of intervention. This incorporates ‘trial and error’ learning, using fit-for-purpose methods to understand the subtleties of the system’s dynamic behaviours [104], and reflexive practices founded on organisational learning principles [76, 89]. Implementation strategies should be loosely specified while attached to clearly defined goals so they can be locally adapted for maximum effect and incorporate ‘bottom up’ expertise and innovatio n[98, 99]. Intervention ‘fidelity’ is thus indicated by the presence of underlying principles, or through the achievement of goals, rather than by faithful adherence to standardised strategie s[64, 201]. Evaluation that informs on-going mobilisation efforts (e.g. developmental evaluation) is warranted [109]. Peters calls this commitment to testing and continuously revising strategies a *“scientific habit of mind”* [97].

## 3. What’s next for advancing systems-informed knowledge mobilisation?

There are several challenges in applying systems thinking to real-world KM practice. First, the evidence-base for the effectiveness of systems thinking in KM is nascent and scattered, in part because systems approaches are diverse and applied very differently [202, 203]. There are calls for further empirical research [204] including in-depth case studies and cross-case analyses, realist research and meta-ethnographie s[3, 126, 134, 138, 205]. Most pressingly, we need to answer the question, ‘*How do we know when systems thinking has enhanced KM?’* But generating high quality systems evidence may require new methodologies and new ways of thinking about health and its interconnections with society and the environment [33, 197, 206]. As Rutter at a l[197]. argue, *“Existing approaches to the generation and use of evidence remain necessary, but are not sufficient”.* The turn to realist, narrative and ethnographic methods in public health reflects this shifting terrain, but we are yet to realise the impacts of this next phase of engaging with complexity [207].

Second, system thinking is conceptually and operationally challenging [62]. It counters many familiar approaches [16] and much of the literature is abstract and theoretically dense [131, 137, 150]. Organisational and individual capacities for using systems theory may be limited [55, 208] and impeded by the often confusing terminology and definitions [46, 204]. Policy partners can experience political pressure to identify simple, easy-to-explain and quick-to-implement solutions [46] using familiar methods [175]. Such challenges may be exacerbated by a lack of professional incentives for cross-sector collaboration [142], perceptions that systems approaches are more resource intensive than traditional methods, and the often longer timeframes they require [187, 195].

Third, there are no universal indicators or standards for good systems-informed KM practice. The KM literature often fails to identify tangible or measurable outcomes, or even to explain the nature of the knowledge that is to be mobilised [2]. There is emerging agreement about the scope and tasks of KM (as illustrated by the archetypes described above [3]) but systems thinking indicates that they are not fixed: KM practices need to be deployed in myriad configurations depending on the local context and goals (see also [31, 142] who identify core skills for transdisciplinary KM). Given the degree to which KM is influenced by power relationships and disciplinary traditions—each sensitive to different indicators and outcomes, and focused on different process paths—what counts as success, for whom and why, is invariably a contentious value-laden issue [17, 85, 209]. Some systems thinkers refute the notion of ‘best practice’ within complex, dynamic contexts – an assertion that might equally apply to KM practices [92, 114]. Thus, while many principles for systems-informed KM may be transferable, a suite of tightly specified practices is unlikely to be applicable in all contexts and circumstances [56, 187].

We make four suggestions for advancing systems-informed knowledge mobilisation:

i. Be specific about what is meant when using the term ‘systems thinking’. What key ideas or methods are included? For what KM purposes? How are they (to be) operationalised? For example, developing systems maps or models is not the same as co-production - they might be done together or separately but with different likely impacts.
ii. Describe counterfactual scenarios in KM so the added value of systems thinking becomes clearer. For example, is a systems approach providing an alternative to a specific model of ‘business-as-usual’ (e.g. one using a linear logic model)? Or is it supplementing (or replacing) another form of collaborative problem solving such as community development?
iii. When evaluating KM, cast the net widely to capture impact. Any intervention which activates the agents in a system will have different effect sizes in different contexts, and may also impact different associated problems [29, 210]. This means that effects and the knowledge employed or generated in their production will take time, care and resources to track thoroughly.
iv. The uncertainty created by complexity is exacerbated when there is poor understanding of how and why change occurred. Use methods that can track KM effectively in complex systems. A key strategy is to make more visible the mechanisms of KM practice, and the short- and long-term impacts of interventions. Careful monitoring and reporting, theory-driven evaluation and process evaluation that utilises qualitative methods can make important contributions to understanding and explaining what occurred [46, 66, 144, 157, 211].

## Conclusion

This paper explores the contribution that systems thinking makes to the planning and implementation of KM in public health policy and practice and suggests areas where there is further potential for value gains. By applying a systems thinking framework to a suite of KM practices, we develop an argument that systems thinking not only enhances but fundamentally transforms KM. It changes what we mean by knowledge, identifying it as a process as much as a transferable product. Knowledge here is pluralistic, informed by multiple parts of the system and reconstituted through use. This contrasts with traditional conceptualisations of exemplary knowledge remaining unchanged through use (fidelity). Systems thinking also changes what we mean by mobilisation – repositioning it as a socially situated, non-prescriptive and potentially destabilising practice that is contingent on addressing power dynamics and on continual adaptive learning about interactions between mobilisation activities and context. KM is no longer conceptualised as a discrete piece of work within wider efforts to strengthen public health, but as integral to and in continual dialogue with those efforts. The challenges presented by systems-informed KM are also very real but, as Braithwaite and colleagues argue, *“we must grapple with the world we actually inhabit, not the one we wish we did”* [144]. We make four suggestions to facilitate constructive debate and further develop empirical evidence about how systems thinking can enhance our capacity to mobilise knowledge for solving complex problems. These include being specific about what is meant by systems thinking and how it has been operationalised; including counterfactual KM scenarios so the added value of systems thinking is clearer; widening the conceptualisations of impact when evaluating KM to capture a range of systems effects; and using evaluation methods that can effectively track KM in complex systems.

Box 1. Glossary of systems terms used in this paper

**Dynamism** – the complex interactions within systems are in constant flux, giving rise to unexpected changes, including unintended and unwelcome intervention outcomes [135, 136].

**Emergence** – parts of the system interact to generate behaviors that are hard to predict and not produced by those parts alone. Emergent change is spontaneous and difficult to control [28, 137–139].

**Feedback** – information that connects elements of a system and is used by that system to regulate itself. Feedback occurs when an output of an activity is fed back into the system as an input. Changes or interventions in the system can create cause-and-effect feedback loops resulting in systems change. Feedback can be positive, resulting in reinforcing and amplifying effects, or negative, resulting in balancing or reducing effects. Modifying feedback has the potential to restructure the system [10, 14, 50, 104].

**Interdependence** – systems comprise interwoven parts—e.g. people, processes and structures—connected by a common purpose. A change in one part can impact, or trigger a response from, other parts [17, 140, 141].

**Self-organisation** – every system changes according to its own attributes or rules of behavior, adapting its structure and function in response to feedback (including KM strategies) [105, 142, 143]. This change process is unpredictable, but not totally random. Current and future patterns of change are strongly influenced by what has happened before [44, 144].

## Data Availability

Not applicable.

## Abbreviation

KM: Knowledge mobilisation

## References

[1] Social Sciences and Humanities Research Council, Framing our direction 2010-2012. Strategic plan., 2008, Government of Canada: Ottowa. Available from: http://www.sshrc-crsh.gc.ca/about-au_sujet/publications/FramingOurDirection_2010-12_final_e.pdf.

[2] Ward V (2016). Why, whose, what and how? A framework for knowledge mobilisers. Evid Policy.

[3] Davies HT, Powell AE, Nutley SM. Mobilising knowledge to improve UK health care: learning from other countries and other sectors–a multimethod mapping study. Health Serv Deliv Res. 2015;3. 26110190

[4] Nutley S, Davies H, Orr K, Nutley S, Russell S, Bain R, Hacking B, Moran C (2016). Knowledge mobilisation: creating, sharing and using knowledge. Knowledge and Practice in Business and Organisations.

[5] Head B, Alford J: Wicked problems: the implications for public management. In Presentation to panel on public management in practice, International Research Society for Public Management 12th Annual Conference; 26-28 March; Brisbane 2008: 26-28.

[6] Lindblom CE. The science of "muddling through". Public Adm Rev. 1959:79–88.

[7] Jones H (2011). Taking responsibility for complexity: How implementation can achieve results in the face of complex problems. Working Paper 330.

[8] Begun JW, Zimmerman B, Dooley K, Mick S, Wyttenbach M (2003). Health care organizations as complex adaptive systems. In Advances in health care organization theory.

[9] Sweeney K, Griffiths F (2002). Complexity and healthcare: an introduction.

[10] Sterman JD (2006). Learning from evidence in a complex world. Am J Public Health.

[11] Lanham HJ, Leykum LK, Taylor BS, McCannon CJ, Lindberg C, Lester RT (2013). How complexity science can inform scale-up and spread in health care: understanding the role of self-organization in variation across local contexts. Soc Sci Med.

[12] Garside R, Pearson M, Hunt H, Moxham T, Anderson R: Preventing obesity using a ‘whole system’ approach at local and community level: PDG1. A report commissioned by NICE Centre for Public Health Excellence. Peninsula Technology Assessment Group (PenTAG), Peninsula Medical School, Universities of Exeter and Plymouth NICE Centre for Public Health Excellence 2010.

[13] Plsek PE, Greenhalgh T (2001). The challenge of complexity in health care. BMJ.

[14] de Savigny D, Taghreed A (2009). Systems thinking for health systems strengthening.

[15] Kickbusch I, Gleicher D. Governance for health in the 21st century: World Health Organization; 2012.

[16] Best A, Holmes B (2010). Systems thinking, knowledge and action: towards better models and methods. Evid Policy.

[17] Cherney A, Head B (2011). Supporting the knowledge-to-action process: a systems-thinking approach. Evid Policy.

[18] OECD, Applications of Complexity Science for Public Policy: New tools for finding unanticipated consequences and unrealized opportunities, Global Science Forum Workshop 5-7 Oct 2008, 2009: Ettore Majorana International Centre for Scientific Culture, Erice, Sicily Available from: http://www.oecd.org/science/sci-tech/43891980.pdf.

[19] Sanderson I (2006). Complexity, 'practical rationality' and evidence-based policy making. Policy Polit.

[20] Holmes BJ, Best A, Davies H, Hunter D, Kelly MP, Marshall M, Rycroft-Malone J (2017). Mobilising knowledge in complex health systems: a call to action. Evid Policy.

[21] Finegood D, Holmes B (2017). Practical strategies to mobilise knowledge in complex systems.

[22] Smith KE, Joyce KE (2012). Capturing complex realities: understanding efforts to achieve evidence-based policy and practice in public health. Evid Policy.

[23] Luke DA, Stamatakis KA (2012). Systems science methods in public health: dynamics, networks, and agents. Annu Rev Public Health.

[24] Leischow SJ, Best A, Trochim WM, Clark PI, Gallagher RS, Marcus SE, Matthews E (2008). Systems thinking to improve the public’s health. Am J Prev Med.

[25] Willis CD, Mitton C, Gordon J, Best A (2012). System tools for system change. BMJ Qual Saf.

[26] Mabry PL, Olster DH, Morgan GD, Abrams DB (2008). Interdisciplinarity and systems science to improve population health: a view from the NIH Office of Behavioral and Social Sciences Research. Am J Prev Med.

[27] Weber EP, Khademian AM (2008). Wicked Problems, Knowledge Challenges, and Collaborative Capacity Builders in Network Settings. Public Adm Rev.

[28] Beckett K, Farr M, Kothari A, Wye L, le May A (2018). Embracing complexity and uncertainty to create impact: exploring the processes and transformative potential of co-produced research through development of a social impact model. Health Res Policy Syst.

[29] Hawe P (2015). Lessons from complex interventions to improve health. Annu Rev Public Health.

[30] Green LW, Ottoson JM, Garcia C, Hiatt RA (2009). Diffusion Theory and Knowledge Dissemination, Utilization, and Integration in Public Health. Annu Rev Public Health.

[31] Murphy K, Wolfus B, Lofters A. From complex problems to complex problem-solving: transdisciplinary practice as knowledge translation. In: Kirst M, Schaefer-McDaniel N, Hwang S, O'Campo P, editors. Converging disciplines: a transdisciplinary research approach to urban health problems: Springer; 2011. p. 111–29.

[32] Meadows D (2008). Thinking in systems: a primer.

[33] Swinburn BA, Kraak VI, Allender S, Atkins VJ, Baker PI, Bogard JR, Brinsden H, Calvillo A, De Schutter O, Devarajan R, et al. The Global Syndemic of Obesity, Undernutrition, and Climate Change: The Lancet Commission report. Lancet. 2019; Available from. 10.1016/S0140-6736(18)32822-8 30700377

[34] Sawin E (2018). The magic of 'multisolving': six principles and practices to unlock cross-sectoral collaboration. Stanford Social Innovation Review.

[35] Sawin E, McCauley S, Edberg S, Mwaura G, Gutierrez MJ, Multisolving at the intersection of health and climate: lessons from success stories, 2018, Climate Interactive. Available from: https://img.climateinteractive.org/wp-content/uploads/2018/02/Multisolving-at-the-Intersection-of-Health-and-Climate-1.pdf.

[36] Markides C (2010). Crossing the Chasm: How to Convert Relevant Research Into Managerially Useful Research. J Appl Behav Sci.

[37] Cairney P, Oliver K. If scientists want to influence policymaking, they need to understand it: The Guardian Guardian Media Group; 2016.

[38] Bowen SJ, Graham ID (2013). From Knowledge Translation to Engaged Scholarship: Promoting Research Relevance and Utilization. Arch Phys Med Rehabil.

[39] Norman CD, Charnaw-Burger J, Yip AL, Saad S, Lombardo C (2010). Designing health innovation networks using complexity science and systems thinking: the CoNEKTR model. J Eval Clin Pract.

[40] Wilson A, Wutzke S, Overs M (2014). The Australian Prevention Partnership Centre: systems thinking to prevent lifestyle-related chronic illness. Public Health Res Pract.

[41] Bammer G. Key issues in co-creation with stakeholders when research problems are complex. Evid Policy. 2019.

[42] Fitzgerald L, Harvey G (2015). Translational networks in healthcare? Evidence on the design and initiation of organizational networks for knowledge mobilization. Soc Sci Med.

[43] Equal Measure | Harder+Company, Cultivating systems leadership in cross-sector partnerships: lessons from the linked learning regional hubs of excellence, 2017, James Irvine Foundation. Available from: http://www.equalmeasure.org/wp-content/uploads/2017/08/Systems-Leadership-Issue-Brief-081017-FINAL.pdf.

[44] Davidson Knight A, Lowe T, Brossard M, Wilson J. A whole new world: funding in complexity: Collaborate, Newcastle University; 2017. Available from: https://marcusjenal.wordpress.com/2017/05/17/a-whole-new-world-funding-in-complexity/.

[45] Kania J, Kramer M, Senge P, The water of systems change, 2018, FSG. Available from: www.fsg.org/publications/water_of_systems_change?utm_source=newsletter&utm_medium=email&utm_content=Read%20the%20article&utm_campaign=20180604waterofsystemschangeall.

[46] Walton M (2016). Expert views on applying complexity theory in evaluation: opportunities and barriers. Evaluation.

[47] Malhi L, Karanfil Ö, Merth T, Acheson M, Palmer A, Finegood DT (2009). Places to intervene to make complex food systems more healthy, green, fair, and affordable. J Hunger Environ Nutr.

[48] Johnston LM, Matteson CL, Finegood DT (2014). Systems science and obesity policy: a novel framework for analyzing and rethinking population-level planning. Am J Public Health.

[49] Reynolds M, Holwell S: Introducing systems approaches. In Systems approaches to managing change: a practical guide. Edited by Reynolds M, Holwell S. Milton Keynes: Springer; 2010: 1-23.

[50] Midgley G (2006). Systemic intervention for public health. Am J Public Health.

[51] Armitage D, Berkes F, Dale A, Kocho-Schellenberg E, Patton E (2011). Co-management and the co-production of knowledge: Learning to adapt in Canada's Arctic. Glob Environ Chang.

[52] Sherriff SL, Miller H, Williamson A, Tong A, Muthayya S, Redman S, Bailey S, Eades S, Haynes A. Building trust and sharing power for co-creation in Aboriginal health research: a stakeholder interview study. Evid Policy. 2019.

[53] Hernandez A, Ruano AL, Marchal B, San Sebastian M, Flores W. Engaging with complexity to improve the health of indigenous people: a call for the use of systems thinking to tackle health inequity. Int J Equity Health. 2017;16. 10.1186/s12939-017-0521-2 PMC5319053 28219429

[54] Abercrombie R, Boswell K, Thomasoo R (2018). Thinking big: how to use theory of change for systems change.

[55] Holmes BJ, Noel K (2015). Time to shift from systems thinking-talking to systems thinking-action: Comment on" Constraints to applying systems thinking concepts in health systems: A regional perspective from surveying stakeholders in Eastern Mediterranean countries". Int J Health Policy Manag.

[56] Best A, Terpstra JL, Moor G, Riley B, Norman CD, Glasgow RE (2009). Building knowledge integration systems for evidence-informed decisions. J Health Organ Manag.

[57] Datta A, Shaxson L, Pellini A (2012). Capacity, complexity and consulting. Lessons from managing capacity development projects.

[58] Zurcher KA, Jensen J, Mansfield A (2018). Using a Systems Approach to Achieve Impact and Sustain Results. Health Promot Pract.

[59] Gates EF (2016). Making sense of the emerging conversation in evaluation about systems thinking and complexity science. Eval Program Plann.

[60] Egan M, McGill E, Penney T, de Cuevas RA, Er V, Orton L, White M, Lock K, Cummins S, Savona N. What to consider when planning a systems evaluation. Guidance on Systems Approaches to Local Public Health Evaluation. Part 2: National Institute for Health Research School for Public Health Research; 2019. Available from.

[61] Sturgiss E, Luig T, Campbell-Scherer DL, Lewanczuk R, Green LA (2019). Using Concept Maps to compare obesity knowledge between policy makers and primary care researchers in Canada. BMC Res Notes.

[62] Freebairn L, Rychetnik L, Atkinson J-A, Kelly P, McDonnell G, Roberts N, Whittall C, Redman S (2017). Knowledge mobilisation for policy development: implementing systems approaches through participatory dynamic simulation modelling. Health Res Policy Syst.

[63] Freebairn L, Atkinson J-A, Osgood ND, Kelly PM, McDonnell G, Rychetnik L (2019). Turning conceptual systems maps into dynamic simulation models: An Australian case study for diabetes in pregnancy. PLoS One.

[64] Hawe P, Shiell A, Riley T (2009). Theorising interventions as events in systems. Am J Community Psychol.

[65] Langeveld K, Stronks K, Harting J (2016). Use of a knowledge broker to establish healthy public policies in a city district: a developmental evaluation. BMC Public Health.

[66] Langlois EV, Becerril Montekio V, Young T, Song K, Alcalde-Rabanal J, Tran N (2016). Enhancing evidence informed policymaking in complex health systems: lessons from multi-site collaborative approaches. Health Res Policy Syst.

[67] Harvey G (2013). The many meanings of evidence: implications for the translational science agenda in healthcare. Int J Health Policy Manag.

[68] McCormack B, Rycroft-Malone J, DeCorby K, Hutchinson AM, Bucknall T, Kent B, Schultz A, Snelgrove-Clarke E, Stetler C, Titler M (2013). A realist review of interventions and strategies to promote evidence-informed healthcare: a focus on change agency. Implement Sci.

[69] Evans S, Scarbrough H (2014). Supporting knowledge translation through collaborative translational research initiatives: ‘Bridging’ versus ‘blurring’ boundary-spanning approaches in the UK CLAHRC initiative. Soc Sci Med.

[70] Bruce A, O'Callaghan K (2016). Inside out: knowledge brokering by short-term policy placements. Evid Policy.

[71] Kitson AL (2009). The need for systems change: reflections on knowledge translation and organizational change. J Adv Nurs.

[72] Cassidy CE, Burgess S, Graham ID (2019). It’s All About the IKT Approach: Three Perspectives on an Embedded Research Fellowship; Comment on “CIHR Health System Impact Fellows: Reflections on ‘Driving Change’ Within the Health System”. Int J Health Policy Manag.

[73] Kislov R, Hodgson D, Boaden R (2016). Professionals as knowledge brokers: The limits of authority in healthcare collaboration. Public Adm.

[74] Lockett A, El Enany N, Currie G, Oborn E, Barrett M, Racko G, Bishop S, Waring J. A formative evaluation of Collaboration for Leadership in Applied Health Research and Care (CLAHRC): institutional entrepreneurship for service innovation. Health Serv Deliv Res. 2014;2. 25642535

[75] Frost H, Geddes R, Haw S, Jackson CA, Jepson R, Mooney JD, Frank J (2012). Experiences of knowledge brokering for evidence-informed public health policy and practice: three years of the Scottish Collaboration for Public Health Research and Policy. Evid Policy.

[76] Senge PM (2006). The fifth discipline: The art and practice of the learning organization.

[77] Bornbaum CC, Kornas K, Peirson L, Rosella LC (2015). Exploring the function and effectiveness of knowledge brokers as facilitators of knowledge translation in health-related settings: a systematic review and thematic analysis. Implement Sci.

[78] Best A, Hiatt RA, Norman CD (2008). Knowledge integration: conceptualizing communications in cancer control systems. Patient Educ Couns.

[79] Northridge ME, Metcalf SS (2016). Enhancing implementation science by applying best principles of systems science. Health Res Policy Syst.

[80] Farrer L, Marinetti C, Cavaco YK, Costongs C (2015). Advocacy for Health Equity: A Synthesis Review. Milbank Q.

[81] Sabatier PA, Weible CM. In: Sabatier PA, editor. The advocacy coalition framework. In Theories of the Policy Process, vol. 2: Westview Press; 2007. p. 189–220.

[82] Kingdon JW (2003). Agendas, alternatives, and public policies (2nd Edition).

[83] Cairney P, Kwiatkowski R (2017). How to communicate effectively with policymakers: combine insights from psychology and policy studies. Palgrave Commun.

[84] Herbert C, Best A (2011). It's a matter of values: partnership for innovative change. HealthcarePapers.

[85] Murphy K, Fafard P (2012). Taking Power, Politics, and Policy Problems Seriously. J Urban Health.

[86] Weyrauch V, Echt L, Suliman S. Knowledge into policy: going beyond ‘context matters’: International Network for the Availability of Scientific Publications; 2016. Available from: http://www.politicsandideas.org/wp-content/uploads/2016/07/Going-beyond-context-matters-Framework_PI.compressed.pdf.

[87] Mayne R, Green D, Guijt I, Walsh M, English R, Cairney P (2018). Using evidence to influence policy: Oxfam’s experience. Palgrave Commun.

[88] Lavis JN, Robertson D, Woodside JM, McLeod CB, Abelson J (2003). Knowledge Transfer Study G: How can research organizations more effectively transfer research knowledge to decision makers?. Milbank Q.

[89] Hummelbrunner R (2015). Learning, systems concepts and values in evaluation: proposal for an exploratory framework to improve coherence. Inst Dev Stud (IDS) Bull.

[90] Chapman S (2004). Advocacy for public health: a primer. J Epidemiol Community Health.

[91] Boswell C, Smith K (2017). Rethinking policy ‘impact’: four models of research-policy relations. Palgrave Commun.

[92] Snowden DJ, Boone ME (2007). A leader's framework for decision making. Harv Bus Rev.

[93] Riley BL, Robinson KL, Gamble J, Finegood DT, Sheppard D, Penney TL, Best A (2015). Knowledge to action for solving complex problems: insights from a review of nine international cases. Health Promot Chronic Dis Prev Can.

[94] Zimmerman B, Reason P, Rykert L, Gitterman L, Christian J, Gardam M (2013). Front-line ownership: generating a cure mindset for patient safety. HealthcarePapers.

[95] Head BW, Alford J (2015). Wicked Problems: Implications for Public Policy and Management. Adm Soc.

[96] Malakellis M, Hoare E, Sanigorski A, Crooks N, Allender S, Nichols M, Swinburn B, Chikwendu C, Kelly PM, Petersen S (2017). School-based systems change for obesity prevention in adolescents: outcomes of the Australian Capital Territory ‘It's Your Move!’. Aust N Z J Public Health.

[97] Peters DH (2014). The application of systems thinking in health: why use systems thinking?. Health Res Policy Syst.

[98] Willis CD, Best A, Riley B, Herbert CP, Millar J, Howland D (2014). Systems thinking for transformational change in health. Evid Policy.

[99] Plsek PE, Wilson T (2001). Complexity, leadership, and management in healthcare organisations. BMJ.

[100] Pieper S (2004). Good to great in healthcare. Healthc Exec.

[101] Joyce A, Ollis D, Kearney S, Leung L, Foenander E (2019). The influence of contextual factors on implementation fidelity in a whole school approach to prevention of violence against women. Health Promot J Aust.

[102] Greenhalgh T, Robert G, Macfarlane F, Bate P, Kyriakidou O (2004). Diffusion of innovations in service organizations: systematic review and recommendations. Milbank Q.

[103] Jenal M (2016). An alternative to a Theory of Change approach. Rumination on systemic economic and social change.

[104] Paina L, Peters DH (2011). Understanding pathways for scaling up health services through the lens of complex adaptive systems. Health Policy Plan.

[105] Best A, Greenhalgh T, Lewis S, Saul JE, Carroll S, Bitz J (2012). Large-System Transformation in Health Care: A Realist Review. Milbank Q.

[106] Rycroft-Malone J, Burton RC, Wilkinson J, Harvey G, McCormack B, Baker R, Dopson S, Graham ID, Staniszewska S, Thompson C (2016). Collective action for implementation: a realist evaluation of organisational collaboration in healthcare. Implement Sci.

[107] Holmes B. Co-producing health research: saying what we mean, meaning what we say, and learning as we go. Michael Smith Foundation for Health Research: Michael Smith Foundation for Health Research; 2017. Available from: http://www.msfhr.org/news/blog-posts/co-producing-health-research?platform=hootsuite. Accessed: 27 Jul 2017.

[108] Laihonen H (2015). A managerial view of the knowledge flows of a health-care system. Knowl Manag Res Pract.

[109] Patton MQ (2011). Developmental evaluation: applying complexity concepts to enhance innovation and use.

[110] Marshall M, Pagel C, French C, Utley M, Allwood D, Fulop N, Pope C, Banks V, Goldmann A (2014). Moving improvement research closer to practice: the Researcher-in-Residence model. BMJ Qual Saf.

[111] Joyce A, Green C, Kearney S, Leung L, Ollis D (2018). Alignment and political will: upscaling an Australian respectful relationships program. Health Promot Int.

[112] Foster-Fishman PG, Nowell B, Yang H (2007). Putting the system back into systems change: a framework for understanding and changing organizational and community systems. Am J Community Psychol.

[113] Snowden D, Cynefin framework introduction. CognitiveEdge, 2010, CognitiveEdge. Available from: https://cognitive-edge.com/videos/cynefin-framework-introduction/. Accessed: 18 Jun 2019.

[114] Ramalingam B, Laric M, Primrose J (2014). From best practice to best fit: understanding and navigating wicked problems in international development.

[115] Fraser SW, Greenhalgh T (2001). Coping with complexity: educating for capability. BMJ.

[116] Alford C (2017). How systems mapping can help you build a better theory of change. In too deep: tools for tackling tough problems.

[117] Funnell SC, Rogers PJ. Purposeful program theory: effective use of theories of change and logic models: Wiley; 2011.

[118] Sniehotta FF, Araújo-Soares V, Brown J, Kelly MP, Michie S, West R (2017). Complex systems and individual-level approaches to population health: a false dichotomy?. Lancet Public Health.

[119] Riley B, Willis C, Holmes B, Finegood D, Best A, McIsaac J, Brownson R, Coldiitz A, Protor E (2018). Systems thinking and dissemination and implementation research. Dissemination and Implementation Research in Health: Translating Science to Practice.

[120] Brandes U, Robins G, McCranie ANN, Wasserman S (2013). What is network science?. Netw Sci.

[121] Bailie R, Matthews V, Brands J, Schierhout G (2013). A systems-based partnership learning model for strengthening primary healthcare. Implement Sci.

[122] Bryson JM, Crosby BC, Stone MM (2015). Designing and Implementing Cross-Sector Collaborations: Needed and Challenging. Public Adm Rev.

[123] Wutzke S, Rowbotham S, Haynes A, Hawe P, Kelly P, Redman S, Davidson S, Stephenson J, Overs M, Wilson A (2018). Knowledge mobilisation for chronic disease prevention: the case of the Australian Prevention Partnership Centre. Health Res Policy Syst.

[124] Boaz A, Locock L, Ward V (2015). Whose evidence is it anyway?. Evid Policy.

[125] Nowotny H, Scott P, Gibbons M (2003). Introduction: 'Mode 2' revisited: the new production of knowledge. Minerva.

[126] Rycroft-Malone J (2015). It's more complicated than that: Comment on "Translating evidence into healthcare policy and practice: single versus multi-faceted implementation strategies - is there a simple answer to a complex question?". Int J Health Policy Manag.

[127] Graham ID, Kothari A, McCutcheon C. Moving knowledge into action for more effective practice, programmes and policy: protocol for a research programme on integrated knowledge translation. Implement Sci. 2018;13. 10.1186/s13012-017-0700-y PMC5797415 29394932

[128] McKibbon KA, Lokker C, Wilczynski NL, Ciliska D, Dobbins M, Davis DA, Haynes RB, Straus SE (2010). A cross-sectional study of the number and frequency of terms used to refer to knowledge translation in a body of health literature in 2006: a Tower of Babel?. Implement Sci.

[129] Greenhalgh T, Wieringa S (2011). Is it time to drop the ‘knowledge translation’ metaphor? A critical literature review. J R Soc Med.

[130] Nutley S, Powell A, Davies H. What counts as good evidence? Provocation paper for the Alliance for Useful Evidence: Alliance for Useful Evidence; 2013.

[131] Rusoja E, Haynie D, Sievers J, Mustafee N, Nelson F, Reynolds M, Sarriot E, Swanson RC, Williams B (2018). Thinking about complexity in health: A systematic review of the key systems thinking and complexity ideas in health. J Eval Clin Pract.

[132] Meadows D. Leverage points: places to intervene in a system: The Sustainability Institute, Vermont; 1999. Available from: http://drbalcom.pbworks.com/w/file/fetch/35173014/Leverage_Points.pdf.

[133] Lich KH, Ginexi EM, Osgood ND, Mabry PL (2013). A call to address complexity in prevention science research. Prev Sci.

[134] Riley B, Norman CD, Best A (2012). Knowledge integration in public health: a rapid review using systems thinking. Evid Policy.

[135] Eppel E, Matheson A, Walton M (2011). Applying Complexity theory to New Zealand public policy: Principles for Practice. Policy Q.

[136] Khan S, Vandermorris A, Shepherd J, Begun JW, Lanham HJ, Uhl-Bien M, Berta W. Embracing uncertainty, managing complexity: applying complexity thinking principles to transformation efforts in healthcare systems. BMC Health Serv Res. 2018;18. 10.1186/s12913-018-2994-0 PMC5863365 29562898

[137] Lissack MR (1999). Complexity: the science, its vocabulary, and its relation to organizations. Emergence.

[138] Cairney P (2012). Complexity Theory in Political Science and Public Policy. Polit Stud Rev.

[139] DeCoste S, Puri J (2019). Complexity, climate change and evaluation: IEU Working Paper No. 02.

[140] Trochim WM, Cabrera DA, Milstein B, Gallagher RS, Leischow SJ. Practical challenges of systems thinking and modeling in public health. Am J Public Health. 2006;96. 10.2105/AJPH.2005.066001 PMC1470516 16449581

[141] Penney LS, Nahid M, Leykum LK, Lanham HJ, Noël PH, Finley EP, Pugh J (2018). Interventions to reduce readmissions: can complex adaptive system theory explain the heterogeneity in effectiveness? A systematic review. BMC Health Serv Res.

[142] Kitson A, Brook A, Harvey G, Jordan Z, Marshall R, O’Shea R, Wilson D (2018). Using complexity and network concepts to inform healthcare knowledge translation. Int J Health Policy Manag.

[143] Reed JE, Howe C, Doyle C, Bell D. Simple rules for evidence translation in complex systems: a qualitative study. BMC Med. 2018;16. 10.1186/s12916-018-1076-9 PMC6009041 29921274

[144] Braithwaite J, Churruca K, Long JC, Ellis LA, Herkes J. When complexity science meets implementation science: a theoretical and empirical analysis of systems change. BMC Med. 2018;16. 10.1186/s12916-018-1057-z PMC5925847 29706132

[145] Chughtai S, Blanchet K (2017). Systems thinking in public health: a bibliographic contribution to a meta-narrative review. Health Policy Plan.

[146] OECD. Systems approaches to public sector challenges: working with change: OECD Observatory of Public Sector Innovation; 2017. Available from: http://www.oecd.org/gov/systems-approaches-to-public-sector-challenges-9789264279865-en.htm.

[147] Allender S, Brown AD, Bolton KA, Fraser P, Lowe J, Hovmand P. Translating systems thinking into practice for community action on childhood obesity. Obes Rev. 2019:1–6. 10.1111/obr.12865 PMC6900082 31359617

[148] Holmes B, Finegood D, Riley B, Best A, Brownson R, Colditz G, Proctor E (2012). Systems thinking in dissemination and implementation research. Dissemination and Implementation Research in Health: Translating Science To Practice.

[149] Carey G, Malbon E, Carey N, Joyce A, Crammond B, Carey A (2015). Systems science and systems thinking for public health: a systematic review of the field. BMJ Open.

[150] Burnes B (2005). Complexity theories and organizational change. Int J Manag Rev.

[151] Martin CM, Félix-Bortolotti M (2010). W(h)ither complexity? The emperor's new toolkit? Or elucidating the evolution of health systems knowledge?. J Eval Clin Pract.

[152] Shankardass K, O'Campo P, Muntaner C, Bayoumi AM, Kokkinen L (2018). Ideas for Extending the Approach to Evaluating Health in All Policies in South Australia Comment on "Developing a Framework for a Program Theory-Based Approach to Evaluating Policy Processes and Outcomes: Health in All Policies in South Australia". Int J Health Policy Manag.

[153] Powell A, Davies H, Nutley S (2017). Missing in action? The role of the knowledge mobilisation literature in developing knowledge mobilisation practices. Evid Policy.

[154] Leykum LK, Parchman M, Pugh J, Lawrence V, Noël PH, McDaniel RR (2010). The importance of organizational characteristics for improving outcomes in patients with chronic disease: a systematic review of congestive heart failure. Implement Sci.

[155] Leykum LK, Pugh J, Lawrence V, Parchman M, Noël PH, Cornell J, McDaniel RR (2007). Organizational interventions employing principles of complexity science have improved outcomes for patients with Type II diabetes. Implement Sci.

[156] Weiss CH (1986). The circuitry of enlightenment: Diffusion of social science research to policymakers. Knowledge.

[157] Bond L, Glover S, Godfrey C, Butler H, Patton GC (2001). Building capacity for system-level change in schools: lessons from the Gatehouse Project. Health Educ Behav.

[158] Patton GC, Bond L, Carlin JB, Thomas L, Butler H, Glover S, Catalano R, Bowes G (2006). Promoting social inclusion in schools: a group-randomized trial of effects on student health risk behavior and well-being. Am J Public Health.

[159] Skarzauskiene A (2010). Managing complexity: systems thinking as a catalyst of the organization performance. Meas Bus Excell.

[160] Kerzner H. Project management: a systems approach to planning, scheduling, and controlling: Wiley; 2017.

[161] Goldberg KI. Crisis decision-making: Understanding the decision-making process during emergencies. CRISIS. 2013;25.

[162] Chandler J, Rycroft-Malone J, Hawkes C, Noyes J (2016). Application of simplified Complexity Theory concepts for healthcare social systems to explain the implementation of evidence into practice. J Adv Nurs.

[163] Reed JE, Green S, Howe C. Translating evidence in complex systems: a comparative review of implementation and improvement frameworks. Int J Qual Health Care. 2018. 10.1093/intqhc/mzy158 PMC6464095 30060185

[164] Nowotny H, Lepenies W (2003). Re-thinking science: from reliable knowledge to socially robust knowledge. In Entangled Histories and Negotiated Universals.

[165] Nutley SM, Walter I, Davies HT. Using evidence: How research can inform public services: Policy press; 2007.

[166] Bristow D, Carter L, Martin S (2015). Using evidence to improve policy and practice: the UK What Works Centres. Contemp Soc Sci.

[167] Nutley S, Boaz A, Davies H, Fraser A (2019). What works now? Continuity and change in the use of evidence to improve public policy and service delivery. Public Money Manag.

[168] Mullett J (2015). Issues of equity and empowerment in knowledge democracy: Three community based research examples. Action Res.

[169] Lomas J (2007). The in-between world of knowledge brokering. BMJ.

[170] Ward V, House A, Hamer S (2009). Knowledge brokering: the missing link in the evidence to action chain?. Evid Policy.

[171] King L, Hawe P, Wise M (1998). Making dissemination a two-way process. Health Promot Int.

[172] Chapman S. Public health advocacy and tobacco control: making smoking history: Wiley-Blackwell; 2008.

[173] Glasziou P, Haynes B (2005). The paths from research to improved health outcomes. BMJ Evid Based Med.

[174] Landry R, Amara N, Lamari M (2001). Climbing the ladder of research utilization: Evidence from social science research. Sci Commun.

[175] Brown C (2012). The policy agora: how the epistemological and ideological preferences of policy-makers affect the development of government policy. Human Welfare.

[176] Liverani M, Hawkins B, Parkhurst JO (2013). Political and institutional influences on the use of evidence in public health policy. A systematic review. PLoS One.

[177] Miller RL, Shinn M (2005). Learning from communities: overcoming difficulties in dissemination of prevention and promotion efforts. Am J Community Psychol.

[178] Castro FG, Yasui M (2017). Advances in EBI development for diverse populations: towards a science of intervention adaptation. Prev Sci.

[179] Haynes A, Brennan S, Redman S, Williamson A, Gallego G, Butow P (2016). Figuring out fidelity: a worked example of the methods used to identify, critique and revise the essential elements of a contextualised intervention in health policy agencies. Implement Sci.

[180] Spiel C, Schober B, Strohmeier D (2018). Implementing Intervention Research into Public Policy—the “I^3^-Approach”. Prev Sci.

[181] Caplan N (1979). 2-Communities theory and knowledge utilization. Am Behav Sci.

[182] Choi BCK, Pang T, Lin V, Puska P, Sherman G, Goddard M, Ackland MJ, Sainsbury P, Stachenko S, Morrison H, Clottey C (2005). Can scientists and policy makers work together?. J Epidemiol Community Health.

[183] Wandersman A, Duffy J, Flaspohler P, Noonan R, Lubell K, Stillman L, Blachman M, Dunville R, Saul J (2008). Bridging the gap between prevention research and practice: The interactive systems framework for dissemination and implementation. Am J Community Psychol.

[184] Butler D (2008). Translational research: crossing the valley of death. Nature.

[185] Shiell A, Hawe P, Kavanagh S. Evidence suggests a need to rethink social capital and social capital interventions. Soc Sci Med. 2018; Available online 8 September 2018. 10.1016/j.socscimed.2018.09.006 30219489

[186] Oliver K, Lorenc T, Innvær S (2014). New directions in evidence-based policy research: a critical analysis of the literature. Health Res Policy Syst.

[187] Hawe P, Bond L, Butler H (2009). Knowledge theories can inform evaluation practice: What can a complexity lens add?. N Dir Eval.

[188] Ferlie E, Crilly T, Jashapara A, Peckham A (2012). Knowledge mobilisation in healthcare: A critical review of health sector and generic management literature. Soc Sci Med.

[189] Black LJ (2013). When visuals are boundary objects in system dynamics work. Syst Dyn Rev.

[190] Oliver K, Kothari A, Mays N (2019). The dark side of coproduction: do the costs outweigh the benefits for health research?. Health Res Policy Syst.

[191] Williamson A, Tait H, El Jardali F, Wolfenden L, Thackway S, Stewart J, O’Leary L, Dixon J: How are evidence generation partnerships between researchers and policy-makers enacted in practice? A qualitative interview study. Health Research Policy and Systems 2019, 17:41. 10.1186/s12961-019-0441-2 PMC6466802 30987644

[192] McCabe KE, Wallace A, Crosland A (2015). A model for collaborative working to facilitate knowledge mobilisation in public health. Evid Policy.

[193] Gollust SE, Seymour JW, Pany MJ, Goss A, Meisel ZF, Grande D (2017). Mutual Distrust: Perspectives From Researchers and Policy Makers on the Research to Policy Gap in 2013 and Recommendations for the Future. INQUIRY: J Health Care Org Provision Financ.

[194] Kislov R, Wilson PM, Knowles S, Boaden R (2018). Learning from the emergence of NIHR Collaborations for Leadership in Applied Health Research and Care (CLAHRCs): a systematic review of evaluations. Implement Sci.

[195] Filipe A, Renedo A, Marston C (2017). The co-production of what? Knowledge, values, and social relations in health care. PLoS Biol.

[196] Heaton J, Day J, Britten N (2016). Collaborative research and the co-production of knowledge for practice: an illustrative case study. Implement Sci.

[197] Rutter H, Savona N, Glonti K, Bibby J, Cummins S, Finegood DT, Greaves F, Harper L, Hawe P, Moore L (2017). The need for a complex systems model of evidence for public health. Lancet.

[198] March JG, Olsen JP (1975). The uncertainty of the past: organizational learning under ambiguity. Eur J Polit Res.

[199] Israel BA, Schulz AJ, Parker EA, Becker AB. Critical issues in developing and following community-based participatory research principles. In: Community-based participatory research for health: Jossey-Bass; 2008. p. 47–62.

[200] Brown JS, Duguid P (1991). Organizational learning and communities-of-practice: toward a unified view of working, learning, and innovation. Organ Sci.

[201] Hawe P, Shiell A, Riley T (2004). Complex interventions: how "out of control" can a randomised controlled trial be?. BMJ.

[202] Hawe P (2015). Minimal, negligible and negligent interventions. Soc Sci Med.

[203] Haynes A, Garvey K, Davidson S, Milat A: What can policymakers get out of systems thinking? Policy partners' experiences of a systems-focused research collaboration in preventive health International Journal of Health Policy and Management In Press. 10.15171/ijhpm.2019.86 PMC7054651 32124590

[204] Wutzke S, Morrice E, Benton M, Wilson A (2016). Systems approaches for chronic disease prevention: sound logic and empirical evidence, but is this view shared outside of academia?. Public Health Res Pract.

[205] Sautkina E, Goodwin D, Jones A, Ogilvie D, Petticrew M, White M, Cummins S (2014). Lost in translation? Theory, policy and practice in systems-based environmental approaches to obesity prevention in the Healthy Towns programme in England. Health Place.

[206] El-Sayed AM, Galea S. Systems science and population health: Oxford University Press; 2017.

[207] Hanlon P, Carlisle S, Hannah M, Reilly D, Lyon A (2011). Making the case for a ‘fifth wave’ in public health. Public Health.

[208] Agyepong IA (2015). “Wood already touched by fire is not hard to set alight”; comment on “Constraints to applying systems thinking concepts in health systems: a regional perspective from surveying stakeholders in Eastern Mediterranean countries”. Int J Health Policy Manag.

[209] Ottoson JM (2009). Knowledge-for-action theories in evaluation: Knowledge utilization, diffusion, implementation, transfer, and translation. N Dir Eval.

[210] Hawe P, Bond L, Ghali LM, Perry R, Davison CM, Casey DM, Butler H, Webster CM, Scholz B (2015). Replication of a whole school ethos-changing intervention: different context, similar effects, additional insights. BMC Public Health.

[211] Martin GP, Ward V, Hendy J, Rowley E, Nancarrow S, Heaton J, Britten N, Fielden S, Ariss S (2011). The challenges of evaluating large-scale, multi-partner programmes: the case of NIHR CLAHRCs. Evid Policy.

